# Plasmablastic Lymphoma Found on Autopsy in a Post-Transplant Patient

**DOI:** 10.7759/cureus.8109

**Published:** 2020-05-14

**Authors:** Syed H Abbas, Geetika Goyal, Mohammad A Noory, Abraham Loo

**Affiliations:** 1 Pathology, Monmouth Medical Center, Long Branch, USA; 2 Pathology, Saint Barnabas Medical Center - Robert Wood Johnson Barnabas Health, Livingston, USA

**Keywords:** plasmablastic lymphoma, transplant

## Abstract

Plasmablastic lymphoma (PBL) is an aggressive lymphoma often seen in immunodeficient patients. It can be a diagnostic challenge given its high-grade appearance and lack of staining for traditional B-cell markers. We present an interesting case of a 65-year-old African-American female who presented to the emergency department (ED) with complaints of progressively worsening weakness, fatigue, and dizziness for one month, and dark-colored urine for three days. The patient's medical history was remarkable for a renal and pancreatic transplant in 2008.

## Introduction

Plasmablastic lymphoma (PBL) is an aggressive type of non-Hodgkin lymphoma formerly considered to be a variant of diffuse large B-cell lymphoma; however, it is currently recognized as a distinct clinical-pathological disorder derived from terminally differentiated B-cells. It was first described by Delecluse et al. in 1997 in HIV-positive individuals with oral mucosal lesions [1]. Cases have since been reported in both immunocompetent individuals and in immunocompromised patients as immunodeficiency-associated lymphoproliferative disorders including post-transplant lymphoproliferative disorders (PTLD) [2]. After a transplant, the risk of lymphoma increases from 20% to 120% compared to the general population. We present a case of PBL found on autopsy in a 65-year-old African-American female following a renal and pancreatic transplant.

## Case presentation

A 65-year-old African-American female presented to the emergency department (ED) of our hospital with complaints of progressively worsening weakness, fatigue, and dizziness for one month. The patient reported mild to moderate squeezing, non-radiating chest pain, and shortness of breath associated with mild activity. She had also noted dark-colored urine for three days preceding her arrival, but no bleeding from any other site. She denied any recent fevers, night sweats, or flu-like symptoms. The patient's medical history was remarkable for a renal and pancreatic transplant in 2008, and she had been on a stable dose of prednisone 5 mg OD (once daily), Tacrolimus 5 mg bd (twice daily), and azathioprine 100 mg OD. The patient had been in her usual state of health prior to the onset of these symptoms. She stated that she had received regular blood tests two months prior, which had been unremarkable.

On arrival to the ED, the patient was initially stable with a blood pressure of 130/85 mm Hg, heart rate of 105 beats per minute, and a respiratory rate of 18 breaths per minute. On physical examination, she seemed in distress but was awake, alert, and oriented. The patient had scleral icterus and skin pallor. No palpable hepatosplenomegaly or lymphadenopathy was noted. The remainder of the physical examination including respiratory and cardiovascular system was unremarkable. Initial laboratory findings showed a white blood cell count (WBC) of 14.7 K/microliter, hemoglobin (Hb) of 5.8 g/dl, platelet count of 264,000 per microliter, and an elevated total and direct bilirubin level of 4.5 g/dl and 0.8 g/dl respectively. The coagulation panel was within normal limits. The patient had a low haptoglobin and elevated lactate dehydrogenase consistent with hemolytic anemia. Urine was grossly red-brown in color, and urinalysis and microscopy were consistent with hematuria and proteinuria. Chest X-ray was unremarkable and EKG showed sinus tachycardia.

The patient's clinical condition worsened rapidly within three hours of arrival to the hospital, and a rapid response was called for respiratory distress and hypotension. On evaluation, the patient was found to be obtunded with a blood pressure of 80/46 mm Hg and a respiratory rate of 40 breaths per minute. A decision was made to electively intubate the patient to protect her airway. Further investigations were ordered including a STAT CT scan, which was negative for any source of bleeding. An arterial blood gas analysis showed metabolic acidosis with respiratory compensation. Repeat labs showed a WBC of 17 K/microliter, Hb of 5.3 g/dl, lactic acid of 8.3 mmol/L, and worsened kidney function with a creatinine of 1.43 mg/dl. The patient was empirically treated for sepsis with fluids and broad-spectrum antibiotics. Within the next 45 minutes, the patient was found to be unresponsive with no palpable pulse. Cardiopulmonary resuscitation was initiated following the advanced cardiac life support (ACLS) protocol. Unfortunately, the return of spontaneous circulation could not be achieved, and resuscitation efforts were stopped as per the family's wishes.

**Figure 1:**
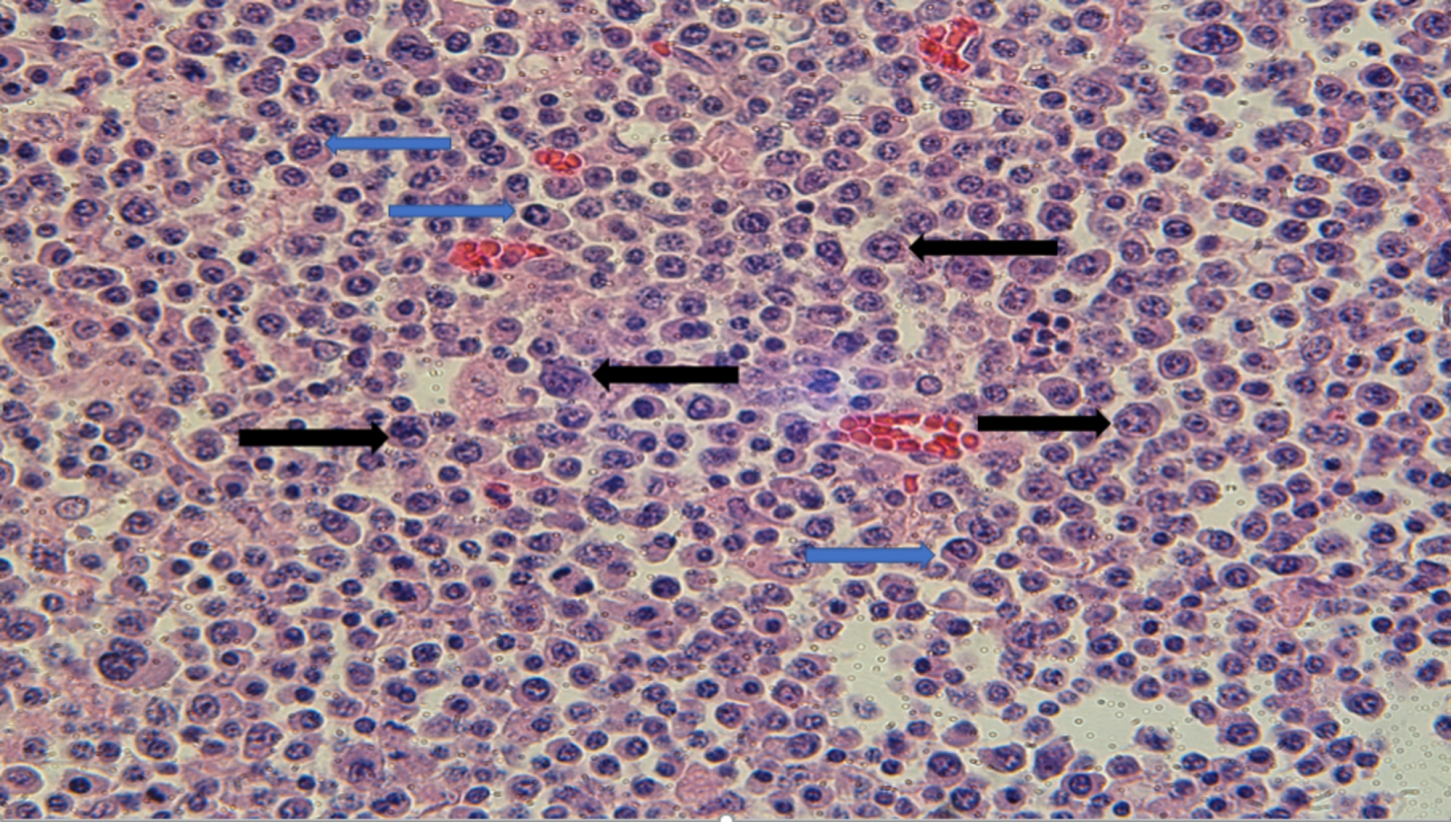
H&E staining of lymph nodes identifying large tumor cells with round nuclei, prominent nucleoli, and coarse chromatin (black arrows); smaller cells with plasmacytic differentiation are also present (blue arrows) H&E: hematoxylin and eosin

An autopsy was performed after appropriate signatures and consent were obtained. On autopsy, multiple lymph nodes were found in the bowel mesentery. Hematoxylin and eosin (H&E)-stained slides of the lymph nodes demonstrated sheets of plasma cells with clock-faced chromatin, perinuclear hof, and nuclear condensation at the periphery. Numerous immunoblasts and PBL cells with plasmacytic differentiation were also identified (Figure 1).

**Figure 2:**
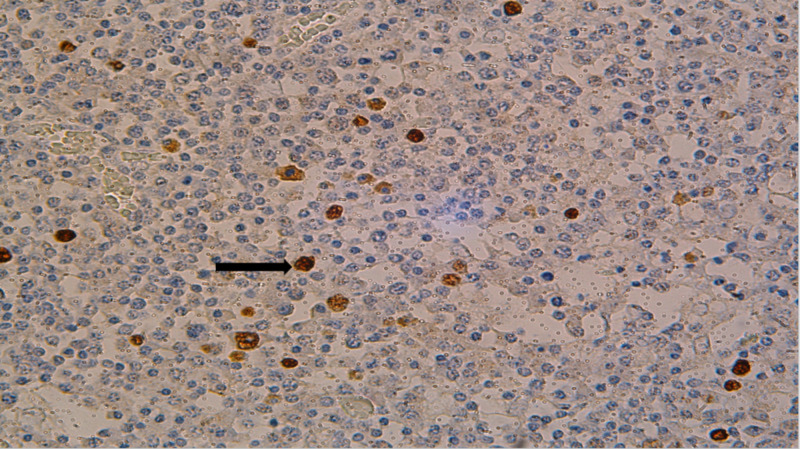
EBER by ISH staining of lymph nodes identifying focal positivity (black arrow) EBER: Epstein-Barr encoding region; ISH: in situ hybridization

The plasma cells and plasmablastic cells were negative for CD20, PAX5, BCL-2, BCL-6, CD10, Cyclin D1, and ALK protein. Multiple cytokeratins, T-cell markers, and S-100 and HMB45 were negative. Epstein-Barr encoding region (EBER) by in situ hybridization (ISH) showed scattered positivity in lymphoma cells (Figure 2).

**Figure 3:**
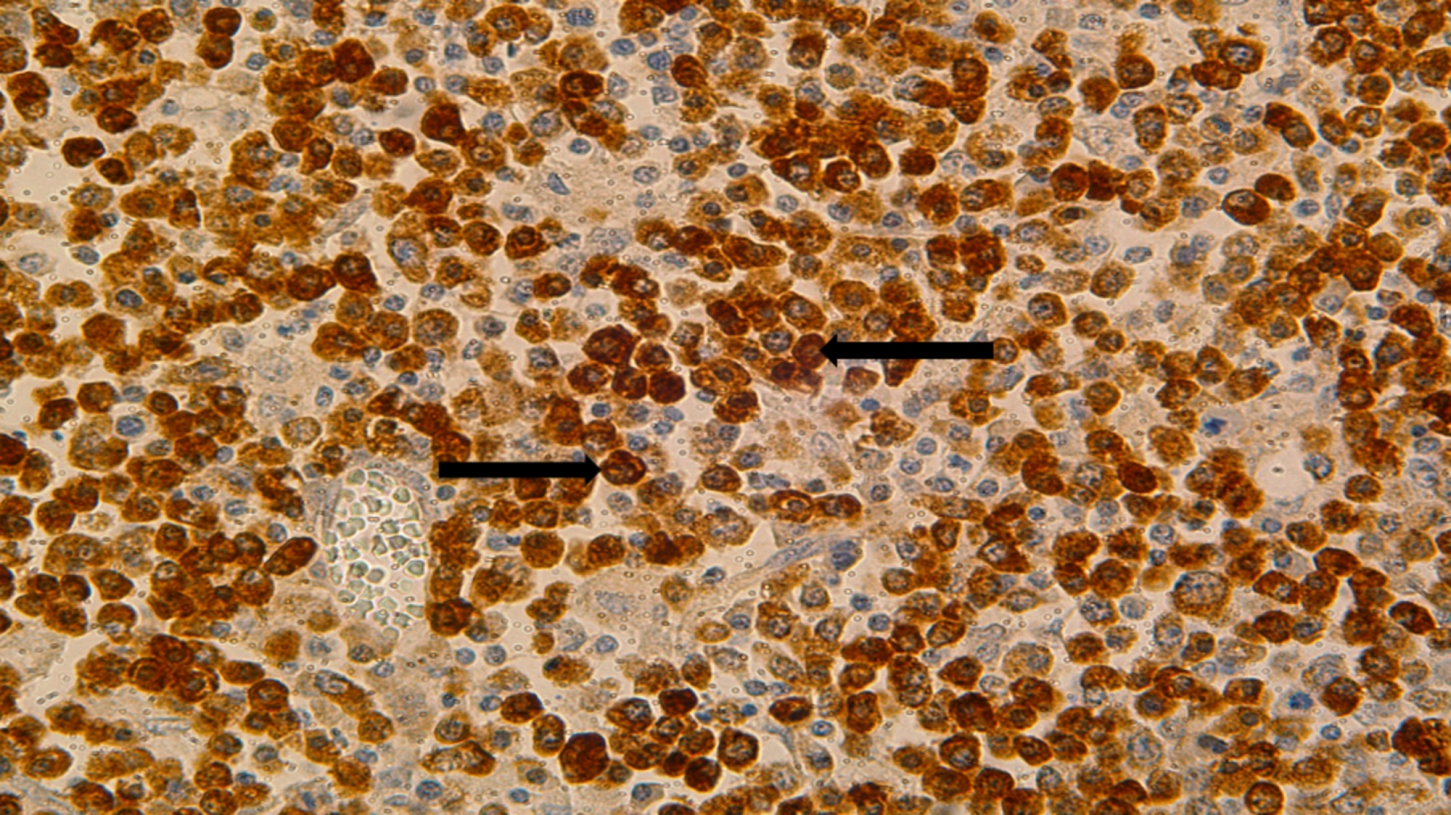
Lambda by ISH staining of lymph nodes shows diffuse positivity (black arrows) ISH: in situ hybridization

**Figure 4:**
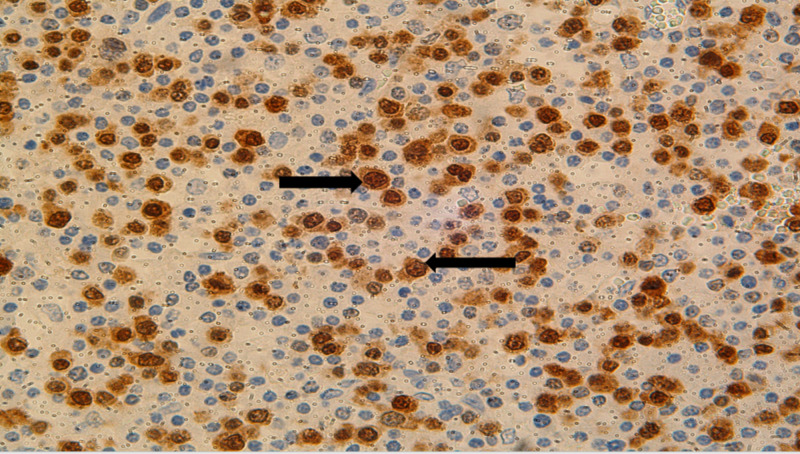
MUM 1 staining of lymph nodes identifying lymphoma cells (black arrows)

Kappa and lambda ISH stains demonstrated an overwhelming predominance of lambda positivity (Figure 3). MUM 1 was diffusely positive in lymphoma cells (Figure 4).

## Discussion

PBL is a large cell lymphoma that can involve the extranodal regions of the head and neck, specifically the oral cavity, in addition to the gastrointestinal tract, genitourinary tract, nasal cavity, paranasal sinuses, skin, bones, central nervous system, liver, lungs, and orbits. Lymph nodes are affected in less than 10% of cases overall but are involved in 30% of post-transplant cases [3]. In our case, the patient had undergone a renal transplant in 2008, and she developed PBL nine years later. Her diagnosis of PBL in the setting of a solid organ transplant qualifies as a PTLD. PTLDs are lymphoid or plasmacytic proliferations that develop as a consequence of immunosuppression in a recipient of a solid organ or stem cell allograft. The incidence of PTLD in adults is dependent on several factors: the type of allograft, Epstein-Barr virus (EBV) infection, and degree of immunosuppression. The overall incidence of PTLD after solid organ transplantation is 1.4-1.7%. The incidence after kidney transplant, heart transplant, and bone marrow transplantation is 0.3-3%, 1.8-9.8%, and 0.4-7.4% respectively. The time to develop PTLD after transplant can vary and typically ranges from 1.5-17 months for renal and heart transplant and up to 101 days after bone marrow transplant. The frequency of PTLD is 10% in all transplant patients with multiple organ transplants. Most cases of PTLD that are EBV-positive develop one to two years after transplant. Most cases of PTLD that are EBV-negative develop three to four years after transplant. Some cases can take more than 10 years, and investigators have introduced a new category for cases with onset of more than 10 years between transplantation and PTLD appearance: very late onset. It represents a distinct clinicopathological subset, occurring more frequently in older patients with a long latency period, EBV- negativity, and with poor response to treatment and worse prognosis [4]. Our case fits in this rare category, as the patient was EBV-negative, developed lymphoma after almost a decade of transplantation, and had a poor outcome.

PBL can histologically range from cells with an immunoblastic appearance to cells with more plasmacytic appearance [5]. The former type is seen in patients infected with HIV and involves the oral, nasal, and paranasal sinus areas. The latter is seen in lymph nodes as well as in extranodal sites and is more common in HIV-negative patients. Immunoblasts have a prominent central nucleolus with variable amounts of cytoplasm. Plasmablastic cells have an eccentric nucleus with a prominent central nucleolus and abundant cytoplasm. In our case, the PBL involved mesenteric lymph nodes and had a plasmacytic differentiation, which is in agreement with the findings in the literature.

The pathogenesis of PBL is not entirely known. It is suggested that deficient cytotoxic T cell function due to pharmacologic immunosuppression in transplantation sensitizes these patients to post-transplant malignancies including PTLD. Recent research has identified recurring rearrangement of MYC and immunoglobulin gene [6,7]. The same rearrangement is also seen in Burkitt lymphoma, but PBL is thought to arise from post-germinal centers as opposed to Burkitt's Lymphoma, which is of germinal center origin. MYC translocation is identified in half of the cases. It may be seen in EBV-positive patients and has been associated with a worse prognosis.

The immunophenotype includes positivity for CD138, CD38, IR4/MUM 1, PRDM1 (also called BLIMP1), XBP1. CD45, CD20, and PAX-5 are either negative or weakly positive in a minority of cells [8]. CD79a is positive in approximately 40% of cases. Cytoplasmic immunoglobulin most frequently expressed is IgG and is either kappa- or lambda-restricted. EMA and CD30 are often expressed and CD56 is positive in 25% of cases. CD10 is positive in 20% of cases. In our case, the cells were negative for B-cell markers and showed light chain restriction on ISH stains. MUM 1 showed diffuse positivity and EBER by in-situ hybridization demonstrated focal positivity in lymphoma cells. All these findings are consistent with a diagnosis of B-cell lymphoma with plasmacytic differentiation. BCL-2, BCL-6, and CD10 stains were negative and were done to rule out follicular lymphoma. Similarly, Cyclin D1 and ALK negativity ruled out mantle cell and anaplastic large cell lymphoma respectively.

Multiple myeloma can appear histologically and immunophenotypically similar to PBL [9]. Clinical correlation may help distinguish between the two. Multiple myeloma will typically manifest with a constellation of findings including lytic lesions, chronic anemia, hypercalcemia, and renal abnormalities. A monoclonal spike is typically detected on serum protein electrophoresis and is typically negative for EBV. Also, multiple myeloma nearly always originates from bone marrow. In our case, a representative section of the marrow taken at autopsy did not have any increased plasma cells. The patient did not have a positive serum protein electrophoresis. The patient also did not have a documented history of monoclonal gammopathy of undetermined significance (MGUS), smoldering myeloma, or multiple myeloma in the medical record.

The prognosis for PBL is poor with a median survival of 6-11 months [10]. The use of highly active antiretroviral therapy (HAART) in HIV-positive patients has shown better overall survival in patients with HIV-associated PBL, but the results have been inconsistent [11]. In some studies, oral plasmablastic has shown a better prognosis compared to extraoral. Advanced stage at diagnosis, bone marrow involvement, no chemotherapy, and age greater than 60 years have been associated with worse prognosis; however, literature research has revealed inconsistent results regarding all the parameters.

## Conclusions

PBL is a rare type of large cell lymphoma that can affect immunocompromised patients such as those who have received a solid organ transplant. Histopathologic examination, immunophenotyping, and clinical history can help distinguish it from other types of lymphoma and malignancy. As post-transplant patients are susceptible to developing post-transplant lymphoproliferative disorders, the physician and the clinical team should keep it in their differential when a patient presents with non-specific findings of weight loss, fever, lethargy, malaise, or lymphadenopathy. In our case, the patient did not have any classic B symptoms, but she did complain of having a generalized weakness for a few days before presenting to the hospital. Also, since 75% of all PTLDs are EBV-positive, a quantitative EBV viral load testing with polymerase chain reaction (PCR) can be done in case of suspicion of PTLD. Serial assays are generally more useful than specific viral load measurements.
